# Emergence of double-dome superconductivity in ammoniated metal-doped FeSe

**DOI:** 10.1038/srep09477

**Published:** 2015-04-01

**Authors:** Masanari Izumi, Lu Zheng, Yusuke Sakai, Hidenori Goto, Masafumi Sakata, Yuki Nakamoto, Huyen L. T. Nguyen, Tomoko Kagayama, Katsuya Shimizu, Shingo Araki, Tatsuo C. Kobayashi, Takashi Kambe, Dachun Gu, Jing Guo, Jing Liu, Yanchun Li, Liling Sun, Kosmas Prassides, Yoshihiro Kubozono

**Affiliations:** 1 Research Laboratory for Surface Science, Okayama University, Okayama 700-8530, Japan; 2 Research Centre of New Functional Materials for Energy Production, Storage and Transport, Okayama University, Okayama 700-8530, Japan; 3 Department of Chemistry, University of Durham, Durham DH1 3LE, UK; 4 Centre for Science and Technology under Extreme Conditions, Osaka University, Osaka 560-8531, Japan; 5 Department of Physics, Okayama University, Okayama 700-8530, Japan; 6 Institute of Physics and Beijing National Laboratory for Condensed Matter Physics, Chinese Academy of Science, Beijing 100190, China; 7 Institute of High Energy Physics, Chinese Academy of Science, Beijing 100049, China; 8 WPI Research Centre, Advanced Institute for Materials Research, Tohoku University, Sendai 980-8577, Japan

## Abstract

The pressure dependence of the superconducting transition temperature (*T*_c_) and unit cell metrics of tetragonal (NH_3_)_y_Cs_0.4_FeSe were investigated in high pressures up to 41 GPa. The *T*_c_ decreases with increasing pressure up to 13 GPa, which can be clearly correlated with the pressure dependence of *c* (or FeSe layer spacing). The *T*_c_ *vs*. *c* plot is compared with those of various (NH_3_)_y_M_x_FeSe (M: metal atoms) materials exhibiting different *T*_c_ and *c*, showing that the *T*_c_ is universally related to *c*. This behaviour means that a decrease in two-dimensionality lowers the *T*_c_. No superconductivity was observed down to 4.3 K in (NH_3_)_y_Cs_0.4_FeSe at 11 and 13 GPa. Surprisingly, superconductivity re-appeared rapidly above 13 GPa, with the *T*_c_ reaching 49 K at 21 GPa. The appearance of a new superconducting phase is not accompanied by a structural transition, as evidenced by pressure-dependent XRD. Furthermore, *T*_c_ slowly decreased with increasing pressure above 21 GPa, and at 41 GPa superconductivity disappeared entirely at temperatures above 4.9 K. The observation of a double-dome superconducting phase may provide a hint for pursuing the superconducting coupling-mechanism of ammoniated/non-ammoniated metal-doped FeSe.

Various metal-intercalated FeSe superconductors have recently been prepared using the liquid NH_3_ technique, and high superconducting transition temperatures, *T*_c_′s, have been reported for NH_3_/metal intercalated FeSe materials, (NH_3_)_y_M_x_FeSe (M: alkali, alkaline-earth and lanthanide atoms)^1^,^2^,^3^,^4^,^5^. Furthermore, we found new superconducting phases in (NH_3_)_y_M_x_FeSe_0.5_Te_0.5_^6^, and their *T*_c_′s were lower than those of the corresponding (NH_3_)_y_M_x_FeSe analogues. The highest reported *T*_c_ in these materials is now 46 K for (NH_3_)_y_Na_0.5_FeSe^1^. Therefore, the (NH_3_)_y_M_x_FeSe materials are attractive research targets in the hunt for high-*T*_c_ superconductors. The *T*_c_ increases markedly with the intercalation of ions with smaller ionic radius^5^. Furthermore, a correlation between *T*_c_ and the lattice constant *c* was observed in (NH_3_)_y_M_x_FeSe materials^5^, suggesting that an increase in two-dimensionality (2D) is a key for raising the *T*_c_ of the materials. This would be similar to HfNCl, in which NH_3_ or tetrahydrofuran (THF) is inserted into the space between HfNCl layers^7^. If the dimensionality is the key factor for raising the *T*_c_ in metal doped FeSe, it is important to investigate the *T*_c_ systematically as a function of *c* in (NH_3_)_y_M_x_FeSe, *i.e.*, the effect of both physical and chemical pressures on *T*_c_ should be comprehensively studied to clarify the correlation between *T*_c_ and plane spacing.

Very recently, we investigated the pressure dependence of *T*_c_ in (NH_3_)_y_Cs_0.4_FeSe up to 3.2 GPa and plotted the *T*_c_ as a function of pressure *p*, which showed a negative pressure effect for *T*_c_^5^. From our previous data^5^, the *T*_c_ *vs*. *p* plot was found to show a brief upward deviation from its straight-line approximation. However, the *T*_c_ has not previously been plotted as a function of *c* or FeSe plane spacing because the pressure dependence of lattice constants has not been available. In the present study we examined the crystal structure of (NH_3_)_y_Cs_0.4_FeSe over the wide pressure range of 0 to 41 GPa using synchrotron X-ray diffraction, and the lattice constants *a* and *c* were determined at each *p*. The *T*_c_ was plotted as a function of *c*. Furthermore, we measured the electrical resistance, *R*, from 1.9 to 41 GPa, and the *T*_c_ value was determined from the *R* *vs.* *T* plot at each *p*; the DC/AC magnetic susceptibility measurement was not performed above 4 GPa since it is difficult in the higher pressure range than 4 GPa. Namely, the resistance is only a realistic tool for proving superconductivity at high pressures. In this report, the *T*_c_ values are plotted as a function of *p* from 0 to 41 GPa, and the pressure range is extended in comparison with that (0–3.2 GPa) in the previous report^5^. It has been found from the *T*_c_ *vs.* *c* plot obtained that the change in *T*_c_ is related to the change in the FeSe plane spacing up to 11 GPa, where the superconductivity vanishes. Surprisingly, the *T*_c_ quickly recovers above 13 GPa and reaches 49 K at 21 GPa, which is one of the highest reported for bulk superconductors of FeSe-derived materials. The *T*_c_ slowly decreases above 21 GPa and at 41 GPa no superconductivity is observed at temperatures above 4.9 K.

## Results

### Pressure dependence of *T*_c_

**Figure 1:**
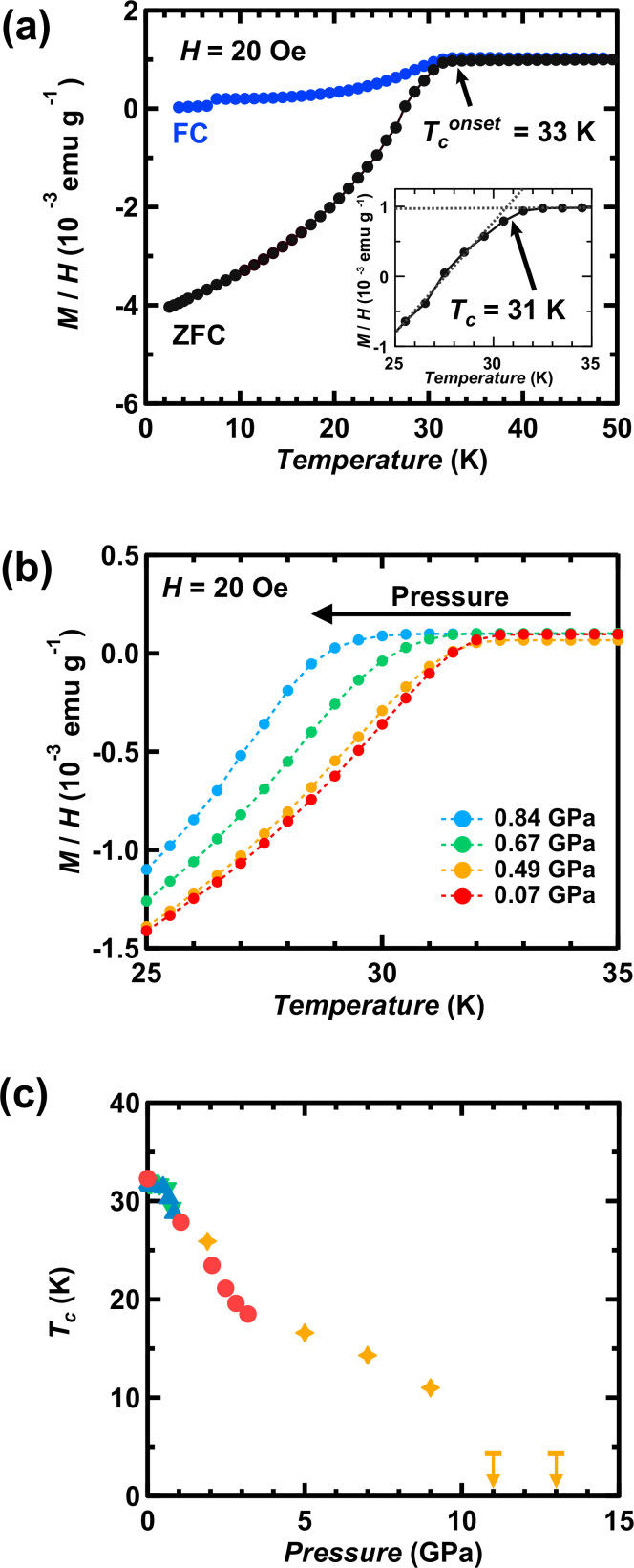
(a) *M*/*H* *vs.* *T* plots for (NH_3_)_y_Cs_0.4_FeSe (ZFC and FC modes) at ambient pressure and (b) *M*/*H* *vs.* *T* plots in ZFC mode under pressure. The inset in (a) shows how the *T*_c_ was determined. (c) *T*_c_ *vs.* *p* plot for (NH_3_)_y_Cs_0.4_FeSe (*p* = 0–13 GPa), determined from the temperature dependence of *M*/*H*, *χ*′ and *R* under pressure. In (c), the *T*_c_′s (blue (*M*/*H* in increasing *p*), green (*M*/*H* in lowering *p*) and red (*χ*′ in increasing *p*)) are determined from *M*/*H* *vs.* *T* and *χ*′ *vs.* *T* plots. The plots are shown in Figure 3(b) of our previous paper^5^. The *T*_c_′s (orange) determined from *R* *vs.* *T* plots are newly plotted in (c). In (c), the arrows indicate *T*_c_′s lower than the temperatures denoted by bars.

Figure 1(a) shows the *M*/*H* *vs.* *T* plots of a typical (NH_3_)_y_Cs_0.4_FeSe sample measured under ambient pressure in zero-field cooling (ZFC) and field-cooling (FC) modes; *M* and *H* refer to magnetization and applied magnetic field, respectively. A clear superconducting transition was observed in both modes, with *T*_c_^onset^ and *T*_c_ of 33 and 31 K, respectively, in ZFC mode, compared with 33 and 32 K, respectively, in FC mode; the *T*_c_ was determined from the intersection of two lines (see inset of Figure 1(a)). No anti-PbO type FeSe phase was observed in the *M*/*H* *vs.* *T* plot; it shows the superconductivity with *T*_c_ = 8 K^8^. The shielding fraction at 2.5 K is 30%. These values are the same as those in the previous report^5^. The *M*/*H* *vs.* *T* plots of the (NH_3_)_y_Cs_0.4_FeSe sample measured at different pressures (0–0.84 GPa) are shown in Figure 1(b). The plots gradually shift to the left in higher pressures, as seen from Figure 1(b).

We determined the *T*_c_′s in the pressure range from 1.9 to 13 GPa from the resistance (*R*) *vs.* *T* plots. All *T*_c_′s determined from the temperature dependences of *M*/*H*, *χ*′ and *R* are plotted as a function of *p* in Figure 1(c); *χ*′ is the real part of AC magnetic susceptibility. In the case of *M*/*H* and *χ*′ measurements, the *T*_c_ was determined from the intersection point, as shown in the inset of Figure 1(a). The *R*- *T* curves below 13 GPa (Figure S1 in Supplementary information) showed the clear drop of *R*, but the zero-resistance could not be observed because of polycrystalline powder sample. No observation of zero-resistance has been reported so far in ammoniated metal doped FeSe^1^,^5^. The drop was assigned to the superconducting transition. For resistance measurement, the *T* corresponding to the midpoint of the *R* *vs.* *T* plot in normal and superconducting states was defined as *T*_c_ in the pressure range from 1.9 to 13 GPa, while above 13 GPa the *T*_c_ was determined from the intersection point; the *T*_c_-determination from the midpoint in the pressure range below 13 GPa is the most reasonable way because of existence of background of semiconducting behaviour as seen from Figure S1. As described later, the *T*_c_ determined from the midpoint follows the *T*_c_ – *p* plot determined from *M*/*H* and *χ*′ measurements (Figure 1(c)), verifying the reasonable determination of *T*_c_. The *T*_c_ decreased monotonically up to 11 GPa, except in the low-pressure range (0–0.8 GPa), as described above. At pressures below 1 GPa, the *T*_c_ rose above the straight line. We previously approximated the *T*_c_ *vs.* *p* plot (0–3.2 GPa) with a linear relationship, but the pressure dependence is not linear in the higher pressure range (see Figure 1(c)). At 11 and 13 GPa, no superconductivity was observed down to 4.3 K.

### Pressure dependence of lattice constants

**Figure 2:**
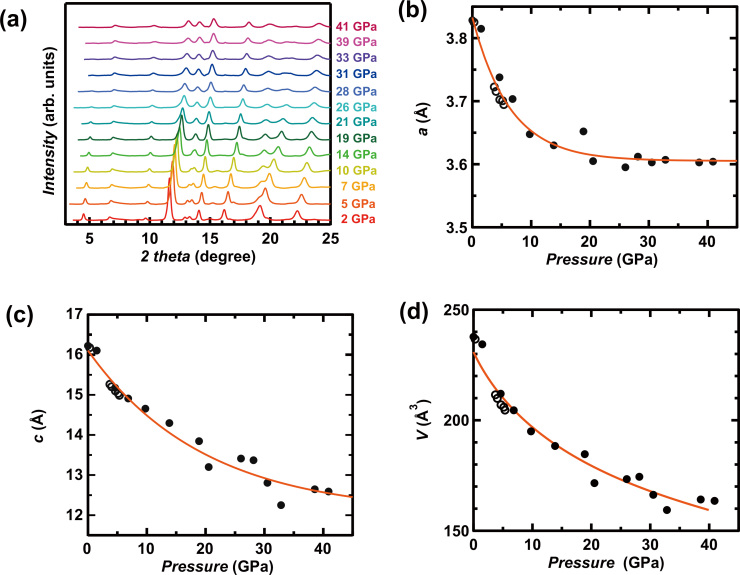
(a) XRD patterns for (NH_3_)_y_Cs_0.4_FeSe under pressure (*p* = 2–41 GPa). (b) and (c) Pressure dependence of *a* and *c* in (NH_3_)_y_Cs_0.4_FeSe. In (b) and (c), the solid and open circles refer to the lattice constants determined from XRD data recorded at the Institute for High Energy physics and SPring-8, respectively. The fitting curves are drawn with a single exponential formula. (d) Pressure dependence of *V* in (NH_3_)_y_Cs_0.4_FeSe is given together with the fitting curve drawn using the semi-empirical third-order Birch-Murnaghan formula.

The XRD patterns measured in the pressure range from 0 to 41 GPa are shown in Figure 2(a); the XRD patterns were recorded at the synchrotron X-ray radiation facility of the Institute of High Energy Physics, Beijing, China, and at SPring-8, Japan. All Bragg reflections gradually shifted to higher angles, implying that the lattice shrinks with increasing pressure. The XRD pattern did not change substantially as pressure was applied to the (NH_3_)_y_Cs_0.4_FeSe sample, indicating no structural transition even across this wide pressure range. The crystal structure of (NH_3_)_y_Cs_0.4_FeSe is body-centred tetragonal (space group: *I*4/mmm) at ambient pressure, and the space group is maintained up to 41 GPa.

The pressure dependence of *a*, *c*, and unit cell volume *V* is shown in Figures 2(b)–(d). The *a* and *c* values were determined by applying LeBail fitting to the XRD patterns. The values determined from XRD patterns recorded at both facilities were substantially consistent. The *a* value decreased dramatically up to 10 GPa and decreased slowly from 10 to 41 GPa, showing an exponential decay of *a*. As seen from Figure 2(c), *c* also decreased monotonically up to 41 GPa. The difference, Δ*a*, between the maximum *a* ( = 3.8331(1) Å) at 0 GPa and the minimum *a* ( = 3.604(2) Å) at 41 GPa was 0.23 Å, and the Δ*a*/*a* (0 GPa) = 0.06. In contrast, the difference, Δ*c*, between the maximum *c* ( = 16.217(1) Å) at 0 GPa and the minimum *c* ( = 12.59(1) Å) at 41 GPa was 3.63 Å, and Δ*c*/*c* (0 GPa) = 0.22. These results suggest that the FeSe layer spacing ( = *c*/2) shrinks easily, which is reasonable considering that the interaction between FeSe layers is due to van der Waals force. Moreover, the compressibility of *c* in (NH_3_)_y_Cs_0.4_FeSe is much higher than that in parent anti-PbO type FeSe^9^. Here, it may be reasonable that the compressibility is smaller in the metal intercalated FeSe than that in pure FeSe because there is additional ionic bonding with metal atoms. Nevertheless, the compressibility is opposite for the expected one. This may be due to the large space maintained by van der Waals force, which is produced by the insertion of ammoniated metal coordinate or NH_3_. Namely, the insertion of molecules leads to the large compressibility. In addition, the anisotropy of compressibility is larger presumably because of the large space between FeSe layers.

The *V* monotonically decreases without any significant change in rate as the pressure increases to 41 GPa, *i.e.*, the *V* – *p* plot can be fitted with semi-empirical third-order Birch-Murnaghan equation^10^ (see Figure 2(d)), showing no structural phase transition; the bulk modulus, *K*_0_, derivative of bulk modulus with respect to *p*, *K*_0_′, and the reference volume (*V* at 0 GPa), *V*_0_, of (NH_3_)_y_Cs_0.4_FeSe are 39.6(1) GPa, 4.7(1) and 232.3(1) Å^3^, respectively. The *K*_0_ is larger than that (*K*_0_ = 30.7(1.1) GPa)^9^ in anti-PbO type FeSe, showing the larger compressibility in (NH_3_)_y_Cs_0.4_FeSe even in volume.

### A correlation between *T*_c_ and structural parameters

**Figure 3:**
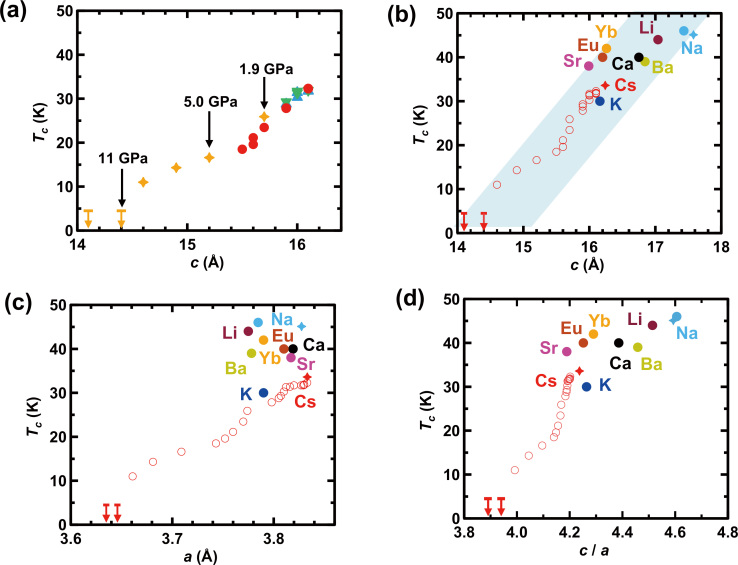
(a) *T*_c_ *vs.* *c* plot evaluated from the plots of *T*_c_ *vs.* *p* (Figure 1(c)) and *c* *vs.* *p* (Figure 2(c)). In (a), symbols' colours correspond to those shown in Figure 1(c). (b) *T*_c_ *vs.* *c* plots constructed by combining the *T*_c_ *vs.* *c* plot (physical pressure effect; Figure 3(a)) and the *T*_c_ *vs.* *c* plot determined from *T*_c_′s and *c*′s for various (NH_3_)_y_M_x_FeSe (chemical pressure effect). (c) *T_c_* *vs.* *a* and (d) *T*_c_ *vs.* *c*/*a* plots constructed based on physical and chemical pressure effects on *T*_c_, which were made the same way as (b). In (b)–(d), open circles refer to the behaviour of *T*_c_ *vs.* lattice constants determined from the pressure dependence of *T*_c_ in (NH_3_)_y_Cs_0.4_FeSe, while solid symbols refer to *T*_c_ *vs.* *c* plots determined with various (NH_3_)_y_M_x_FeSe samples (solid open circles and stars are taken from Refs. 1 and 5, respectively). In (b)–(d), the arrows indicate *T*_c_′s lower than the temperatures denoted by bars.

Figure 3(a) shows a *T*_c_ *vs.* *c* plot constructed based on the plots of *T*_c_ *vs.* *p* (Figure 1(c)) and *c* *vs.* *p* (Figure 2(c)). The *T*_c_ decreases monotonically and reaches 11 K at *c* = 14.6(5) Å (9 GPa) and <4.3 K at *c* = 14.4(5) Å (11 GPa); the *c* values at 9 and 11 GPa are evaluated based on the fitting curve with exponential formula shown in Figure 2(c); the fitting for experimental *c* – *p* plot with simple exponential expression was achieved to evaluate the *c* at any pressure, which was a reasonable way to evaluate the *c* values at the whole pressure range of 0–41 GPa. Figure 3(a) suggests a clear correlation between *T*_c_ and *c*, *i.e.* plane spacing, in which the FeSe plane spacing is *c*/2. On the other hand, the *T*_c_ ( = 31 K) of K_0.5_FeSe with a *c* of 14.0367(7) Å^11^ at 0 GPa is higher than the *T*_c_ ( = 11 K) of (NH_3_)_y_Cs_0.4_FeSe with a *c* of 14.6(5) Å (9 GPa), indicating that the variation of *T*_c_ with *c* (or plane spacing) is not universally consistent for all metal-doped FeSe solids (non-ammoniated and ammoniated solids).

Figure 3(b) shows a *T*_c_ *vs.* *c* plot that includes both the *T*_c_ *vs.* *c* plots obtained from the pressure effect ((NH_3_)_y_Cs_0.4_FeSe) and from the chemical pressure effect (or *T*_c_ *vs.* *c* in various metal-doped FeSe); x is 0.4 or 0.5 in the (NH_3_)_y_M_x_FeSe used to determine the chemical pressure effect. The graph clearly shows that the *T*_c_ is related to *c* (FeSe plane spacing), over all the *c* values recorded, based on both chemical and physical pressure effects. In the (NH_3_)_y_M_x_FeSe solids, the smallest *c* realized at 0 GPa is 14.84(1) Å for (NH_3_)_0.37_K_0.6_Fe_2_Se_2_^3^ (*T*_c_ = 30 K), which is smaller than that (16.16(5) Å)^1^ for ammoniated KFe_2_Se_2_ ( = (NH_3_)_y_K_0.5_FeSe) with *T*_c_ = 30 K; this result (*T*_c_ = 30 K for 14.84(1) Å) is not plotted in the graph shown in Figure 3(b) because the x ( = 0.6/2 = 0.3) is smaller than 0.5. The result does not follow the *T*_c_ – *c* scenario shown in Figure 3(b). The reason why an outlier (*T*_c_ = 30 K for 14.84(1) Å) is obtained in (NH_3_)_y_K_x_FeSe is unclear, but the factor other than *c* may be necessary to be considered. Except for the result, the *T*_c_ – *c* plots for (NH_3_)_y_M_x_FeSe (x ~ 0.5) seem to be meaningful, as seen from Figure 3(b). In addition, a *c* of 14.4 Å in (NH_3_)_y_M_x_FeSe has never been achieved without applying pressure. Thus, we determined the *T*_c_ for an FeSe plane spacing that cannot be achieved without the application of pressure.

Furthermore, we tried to relate *T*_c_ to other factors such as *a*, *c*/*a*, the Se-Fe-Se angle (*α*), and the anion height (height of Se from the Fe plane). As seen from Figure 3(c), the *T*_c_ for (NH_3_)_y_Cs_0.4_FeSe under pressure is related to the lattice constant *a*, *i.e*. the *T*_c_ decreases monotonically with decreasing *a*. However, the *T*_c_ values (chemical pressure effect) realized for various (NH_3_)_y_M_x_FeSe at 0 GPa do not follow the *T*_c_ *vs.* *a* relationship obtained by the application of pressure (physical pressure effect), indicating that the *T*_c_ is not simply or always related to *a*. Moreover, the behaviour of *T*_c_ – *c*/*a* is also different between physical and chemical pressure effects as seen from Figure 3(d).

Since we did not obtain the Rietveld refinements for XRD patterns, but used LeBail fitting, the atomic coordinates of Fe and Se were not obtained. It should be noted that Fe and Se occupy the positions (0.0, 0.5, 0.25) and (0.0, 0.0, z), respectively, and the *α* and anion height cannot be exactly determined. At the present stage, we tried to evaluate *α* and anion height based on the lattice constants at each *p* and the atomic coordinates determined at ambient pressure^5^. The correlations between *T*_c_ and *α* or anion height were investigated over the pressure range of 0 to 13 GPa. The *T*_c_ decreased monotonically when the *α* and anion heights approached the ideal values, 109.5° and 1.38 Å (Figures S2 and S3 in Supplementary Information), respectively, showing different *T*_c_ behaviour from the FeAs materials, in which the *T*_c_ increases when *α* and anion height are close to the ideal values of 109.5° and 1.38 Å, respectively (inverse V-shaped behaviour)^12^,^13^. These results imply that the *T*_c_ may not be simply related to *α* and the anion height, as in FeAs. However, the Rietveld refinement for the XRD pattern at each *p* is absolutely required to clarify more precisely the relationship between *T*_c_ and *α* or the anion height. The relationship between *T*_c_ and anion height is briefly dealt with in the Discussion section, and an interesting scenario is suggested.

### The rapid increase in *T*_c_ above 13 GPa

**Figure 4:**
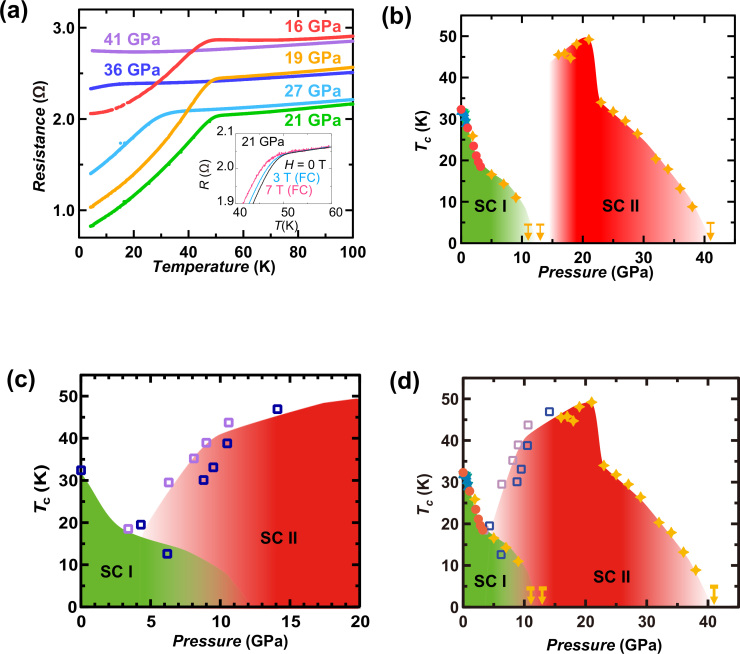
(a) *R* *vs.* *T* plots for (NH_3_)_y_Cs_0.4_FeSe at pressures of 16–41 GPa. Inset of (a): *R* *vs.* *T* plots for (NH_3_)_y_Cs_0.4_FeSe at 21 GPa at 0, 3 and 7 T. (b) *T*_c_ *vs.* *p* plot (*p* = 0–41 GPa) determined from the temperature dependence of *M*/*H*, *χ*′ and *R* for (NH_3_)_y_Cs_0.4_FeSe (sample 1 (first batch)) under pressure. Colours of plots in the pressure range below 13 GPa correspond to those in Figure 1(c). The star refers to the *T*_c_ determined from the *R* *vs.* *T* plot. (c) *T*_c_ *vs.* *p* plot (*p* = 0–14 GPa) determined from the temperature dependence of *R* for (NH_3_)_y_Cs_0.4_FeSe (sample 2 and 3 (second batch)) under pressure. Colours of plots in blue and purple refer to the samples 2 and 3, respectively. (d) *T*_c_ *vs.* *p* plot (*p* = 0–41 GPa) constructed by combining the *T*_c_ *vs.* *p* plots shown in Figures 4(b) and (c). In (b) and (d), the arrows indicate *T*_c_′s lower than the temperatures denoted by bars.

We applied more than 13 GPa pressure to the (NH_3_)_y_Cs_0.4_FeSe sample. Figure 4(a) shows the *R* *vs.* *T* plots from 16 to 41 GPa, which surprisingly show a drop in *R* above 45 K in the limited pressure range from 16 to 21 GPa. The *R* *vs.* *T* plots at 21 GPa were measured in an *H* range of 0 to 7 T (Inset of Figure 4(a)), showing that the *T*_c_ shifted to the left as *H* increased. Therefore, the drop in *R* (Figure 4(a) can be unequivocally assigned to a superconducting transition. The *T*_c_ was plotted as a function of *p* over the wide range of 0 to 41 GPa (Figure 4(b)). As described in the previous section, the *T*_c_ in (NH_3_)_y_Cs_0.4_FeSe decreased monotonically with increasing pressure up to 13 GPa, where superconductivity vanished, but the superconductivity quickly recovered above 13 GPa, and the *T*_c_ became as high as 46 K at 16 GPa. The *T*_c_ increased slightly at pressures up to 21 GPa, reaching 49 K at 21 GPa. The *T*_c_ slowly dropped above 21 GPa, and at 41 GPa no superconductivity was observed down to 4.9 K. This behaviour is similar to that reported previously for Tl_0.6_Rb_0.4_Fe_1.67_Se_2_ and K_0.8_Fe_1.7_Se_2_^14^, in which the *T*_c_′s increased dramatically up to 48 K or 48.7 K from 0 K (~10 GPa) under pressures greater than 11 GPa. The first and the second dome-like superconducting phases were named as SC I and SC II, respectively, as seen from Figure 4(b). Figure 4(b) shows the disappearance of SC I and the emergence of SC II at 11–15 GPa.

To confirm the presence of SC II, we measured the pressure dependence of *T*_c_ for another batch (a second batch) of (NH_3_)_y_Cs_0.4_FeSe, which was made using the same technique as the sample whose results appear in the graph shown in Figure 4(b). This sample is referred to as ‘the second sample’ for the convenience of readers. The *T*_c_ decreased as increasing pressure was applied to the sample. The behaviour of *T*_c_ in the second sample (Figure 4(c)) was the same as the data described above (Figure 4(b)) in the pressure range up to 6 GPa, but the *T*_c_ rapidly increased up to 30 K at 8.8 GPa (see Figure S4 of Supplementary Information). The *R* *vs.* *T* plot for ‘the third sample’ taken from the second batch of material was also measured, and the *T*_c_ *vs.* *p* plot is shown in Figure 4(c). Figure 4(d) shows the *T*_c_ – *p* plots determined from all the (NH_3_)_y_Cs_0.4_FeSe samples. Here it should be noticed that for the samples 2 and 3 of Figures 4(c) and (d), the *T*_c_^mid^′s are plotted for the superconducting transition ascribable to the SC I, while the *T*_c_′s are plotted for that ascribable to SC II, so that the plots can be reasonably compared with those of sample 1.

The *T*_c_ values for the second and the third samples (second batch) are consistent with each other (Figure 4(c)). As seen from Figures 4(b) and (d), the SC II emerged suddenly above 15 GPa in the first batch. On the other hand, as seen from Figures 4(c) and (d), the SC II slowly appeared above 5 GPa in the second batch. The *T*_c_ for the SC II in the second sample reached 47 K at 14 GPa, which is the same as the *T*_c_ for the sample 1 providing the graph shown in Figure 4(b). These results strongly support the presence of SC II.

Here, we briefly discuss the difference in physical and chemical properties of the sample batches, 1 and 2, of (NH_3_)_y_Cs_0.4_FeSe. The *T*_c_ of the second batch was 32.5 K at ambient pressure, and the shielding fraction was 23% at 10 K, showing the same magnetic properties as the other (NH_3_)_y_Cs_0.4_FeSe sample prepared in this study. The XRD of the second batch shows the similar pattern to that reported previously for (NH_3_)_y_Cs_0.4_FeSe^5^, and the Rietveld refinement for the XRD pattern provided the lattice constants (*a* = 3.8232(3) Å and *c* = 16.247(3) Å) which are close to those (*a* = 3.8331(1) Å and *c* = 16.217(1) Å) in Ref. 5. Furthermore, the stoichiometry of Cs was determined by the Rietveld refinement, and the chemical composition for the second batch was evaluated to be (NH_3_)_y_Cs_0.294(2)_FeSe, which is almost the same as that, (NH_3_)_y_Cs_0.255(5)_FeSe, reported previously^5^. These results imply that the second batch is a typical (NH_3_)_y_Cs_0.4_FeSe sample used in this study. Thus, the difference in physical and chemical properties between batches 1 and 2 seems to be too small. Nevertheless, unknown physical and chemical difference may be hidden, but it is unclear at the present stage. More detailed work is indispensable for clarifying the difference.

We carefully checked the *R* *vs.* *T* plots of the second sample at 8.8 GPa (9.5 GPa) to clarify whether the material with a superconducting transition lower than 30 K (33 K) was observed. The *R*- *T* plots at 8.8 and 9.5 GPa showed a very small drop in *R* below 10 K together with the rapid drops at 30 and 34 K, respectively (Figure S4 in Supplementary Information), suggesting the multiple superconducting phases of low and high *T*_c_ at around the pressure range of 8.8 to 9.5 GPa; this may be due to the presence of microstructure corresponding to two superconducting phases, but the exact origin why the multiple superconducting phases were observed in another batch remains to be solved. The investigation using the second batch unequivocally evidenced the presence of second-dome superconducting phase.

These results show the presence of a high-*T*_c_ phase in (NH_3_)_y_Cs_0.4_FeSe (*T*_c_ > 45 K) that emerges in the pressure range from 14 to 21 GPa. The *T*_c_ of 49 K observed at 21 GPa is one of the highest reported in bulk superconductors of FeSe-derived materials. The low-*T*_c_ phase disappears completely at around 11–13 GPa. Therefore, that pressure may be recognized as a ‘quantum critical point’. With reducing pressure, the *T*_c_ follows the *T*_c_ *vs.* *p* plot constructed for increasing pressure (not shown), implying that the pressure dependence of *T*_c_ is reversible.

## Discussion

In this study, it has been found that the *T*_c_ of (NH_3_)_y_Cs_0.4_FeSe is related to the lattice constant *c*, *i.e.* the FeSe plane spacing in the pressure range up to 13 GPa. The variation of *T*_c_ due to plane spacing is convincingly shown not only by the pressure dependence of *T*_c_ in (NH_3_)_y_Cs_0.4_FeSe, but also from the *T*_c_′s in various ammoniated metal doped FeSe's, (NH_3_)_y_M_x_FeSe. Both chemical and physical pressure effects have made clear the dependence of *T*_c_ on FeSe plane spacing. This implies that an increase in 2D nature produces higher *T*_c_′s in (NH_3_)_y_M_x_FeSe, meaning that an increase in Fermi nesting can stabilize the superconducting state. We suggested previously^5^ that a spin-density wave (SDW) ground state with a weak magnetic moment played an important role in superconductivity, as suggested in NaFe_1−x_Co_x_As^15^. Recently, the intercalation of metal atoms into FeSe was achieved using a solvent (ethylenediamine) other than liquid NH_3_, which expands the FeSe plane spacing more than NH_3_^16^. Nevertheless, the *T*_c_ saturates at around 45 K, meaning that *T*_c_ will not rise above 45 K even if the 2D nature increases further.

Furthermore, we applied pressures of more than 13 GPa to (NH_3_)_y_Cs_0.4_FeSe. The new superconducting phase exhibiting higher *T*_c_ emerged dramatically after the first superconducting phase vanished. The re-emerged superconducting phase showed almost a constant *T*_c_ (46–49 K) in the pressure range from 14 to 21 GPa. This behaviour of pressure-induced re-appearance of superconductivity is quite similar to that of previously discovered Tl_0.6_Rb_0.4_Fe_1.67_Se_2_ and K_0.8_Fe_1.7_Se_2_^14^. Our study reveals the presence of a pressure-induced high-*T*_c_ phase in M_x_FeSe. Sun *et al*. suggested that the re-emergence of superconductivity in pressurised Tl_0.6_Rb_0.4_Fe_1.67_Se_2_ and K_0.8_Fe_1.7_Se_2_ is closely associated with a quantum critical transition^16^, in which both the antiferromagnetic phase and the low-*T*_c_ superconducting phase disappear. On the other hand, we observed the multiple superconducting phases of low-*T*_c_ and high-*T*_c_ in the second sample (second batch) of (NH_3_)_y_Cs_0.4_FeSe (see Figures S4 in Supplementary Information) at pressures below 13 GPa. This implies that there are inhomogeneous parts of low-*T*_c_ and high-*T*_c_ phases even at the same pressure.

The different *T* – *p* phase diagram between two batches of (NH_3_)_y_Cs_0.4_FeSe, that the first batch provides the distinguished SC I and SC II while the second batch provides the superposed SC I and SC II, must be discussed. As described in the **results** section, the chemical and physical characters of both batches were almost the same. Therefore, the difference may not be assigned to the different physical/chemical nature. Under high pressure, SC II exactly appeared in both batches, implying that SC II is stabilized under high pressure, while SC I disappeared with increasing pressure. In the intermediate pressure, SC I and SC II seem to coexist in the samples 2 and 3. If the inhomogeneity of applied pressure is present, the SC I and SC II may coexist in the DAC cell. If it is the case, the sample 1 may provide the exact *T* – *p* phase diagram in (NH_3_)_y_Cs_0.4_FeSe, because the complete disappearance of SC I causes the emergence of SC II. The origin is not still clear, but the presence of high-*T*_c_ phase (SC II) was convinced (Figures 4(b)–(d)).

Here, it should be noted that non-ammoniated Tl_0.6_Rb_0.4_Fe_1.67_Se_2_ and K_0.8_Fe_1.7_Se_2_ may belong to another type of alkali selenide superconductor with antiferromagnetic insulating and superconducting phases at ambient pressure; antiferromagnetic insulating phase is not unambiguously found in (NH_3_)_y_Cs_0.4_FeSe. If the antiferromagnetic state is contained in the sample, the deficiency of Fe may be observed. The deficiency of Fe has not been confirmed in ammoniated metal doped FeSe through our study, which was performed by energy dispersive x-ray spectrometry. Thus, the chemical composition of (NH_3_)_y_Cs_0.4_FeSe is different from the above single crystals (Tl_0.6_Rb_0.4_Fe_1.67_Se_2_ and K_0.8_Fe_1.7_Se_2_) concerning Fe deficiency, implying that the average structure of (NH_3_)_y_Cs_0.4_FeSe is not 245 phase (antiferromagnetic order phase)^17^. Since Tl_0.6_Rb_0.4_Fe_1.67_Se_2_ and K_0.8_Fe_1.7_Se_2_ can be expressed as the 245 phase^17^ which was assigned to antiferromagnetic order phase, the volume fraction of the superconducting phase which was assigned to 278^19^ may be lower in the above crystals than that of ammoniated metal doped FeSe. Therefore, considering the different microstructures of (NH_3_)_y_Cs_0.4_FeSe and Tl_0.6_Rb_0.4_Fe_1.67_Se_2_ (or K_0.8_Fe_1.7_Se_2_), the mechanism of emergence of a high-*T*_c_ phase in (NH_3_)_y_Cs_0.4_FeSe would be different from that in non-ammoniated alkali selenide superconductors^18^. Namely, in the former, the relationship between the re-emergence of SC II and magnetic properties is still ambiguous, although the latter was closely associated with the magnetic transition (antiferromagnetic to paramagnetic)^18^.

The pressure dependence of the XRD pattern showed no clear structural phase transition up to 41 GPa, suggesting that the crystal structure of the high-*T*_c_ phase is the same as that of the low-*T*_c_ phase. Here we must stress that detailed information on atomic coordinates has not yet been obtained under pressure, since we achieved only LeBail fitting for XRD patterns up to 41 GPa. This implies that the possibility of changed atomic coordinates in the high-*T*_c_ phase observed above 13 GPa cannot be ruled out.

We present a scenario for the pressure dependence of *T*_c_ on the anion height. Recently, Lu *et al*. reported a V-shaped relationship (V-shaped curve) between *T*_c_ and anion height in FeSe-derived superconductors^19^. Examination of the plot of *T*_c_ *vs.* anion height in FeSe-derived superconductors (Figure 4 of Ref. 18), suggests that the *T*_c_ should decrease when the anion height decreases to 1.45 Å from 1.53 Å (the anion height in (NH_3_)_y_Cs_0.4_FeSe at 0 GPa) and it should increase steeply with a decrease in anion height to less than 1.45 Å^18^. Furthermore, the *T*_c_ should decrease below 1.38 Å (Figure 45 of Ref. 15) since an inverse V-shaped curve is found in FeAs-derived superconductors^12^,^13^. We plotted the *T*_c_ of (NH_3_)_y_Cs_0.4_FeSe as a function of anion height over the wide pressure range of 0–41 GPa (Figure S5 in Supplementary Information), which shows V-shaped/inverse V-shaped behaviour although the turning points of anion height (1.33 and 1.26 Å) of (NH_3_)_y_Cs_0.4_FeSe deviate from the above values (1.45 and 1.38 Å). Thus, a scenario based on V-shaped and inverse V-shaped curves^12^,^13^,^19^ describing the dependence of *T*_c_ on the anion height found in FeSe and FeAs based superconductors may provide a physical basis to explain the pressure dependence of *T*_c_ observed in this study. To verify this interpretation, more precise analyses of XRD patterns with Rietveld refinement are indispensable.

The possibility that a magnetic transition (variation of magnetic structure) produces the re-emergence of superconductivity (or the appearance of a high-*T*_c_ phase) as in Tl_0.6_Rb_0.4_Fe_1.67_Se_2_ and K_0.8_Fe_1.7_Se_2_ cannot completely be ruled out in (NH_3_)_y_Cs_0.4_FeSe at the present. To clarify the correlation between superconductivity and magnetism, measurements of neutron diffraction and magnetism under pressure are indispensable.

Finally, it is important to discuss whether the superconducting coupling mechanism in the high-*T*_c_ superconducting phase is the same as that in the low-*T*_c_ phase. At the present stage, how the second phase is engendered by pressure is still unclear, although a geometrically based scenario is suggested by the empirical results of this study. The fact that the high-*T*_c_ phase appears in (NH_3_)_y_M_x_FeSe as well as M_x_FeSe at high pressure suggests that a pressure-induced high-*T*_c_ phase may be an intrinsic and universal feature in metal doped FeSe superconductors.

## Methods

### Sample preparation and characterizations

The samples of (NH_3_)_y_Cs_0.4_FeSe were prepared according to the method described in the previous paper^5^. The XRD pattern of the sample under pressure was measured at 297 K at two synchrotron radiation facilities, the 4W2 beamline at the Beijing Synchrotron Radiation Facility and BL02B2 at SPring-8. A diamond anvil cell (DAC) was used for the high-pressure XRD measurement. The pressure was determined by monitoring ruby fluorescence. The superconductivity of the (NH_3_)_y_Cs_0.4_FeSe sample was checked by DC magnetic susceptibility (*M*/*H*) recorded by SQUID magnetometer (Quantum Design MPMS2). The *T*_c_′s at 0 GPa for all the samples used in this study were 31 to 32 K as shown in Ref. 5. The pressure dependence of *T*_c_ in the low pressure range (0–3.2 GPa) was determined from the DC (*M*/*H*) and AC (*χ*′) magnetic susceptibilities measured in our previous study^5^, but the *M*/*H* *vs.* *T* plots at low pressures (Figure 1(b)) are presented for the first time in this paper. The difference in *T*_c_ determined from *M*/*H* and *χ*′ of (NH_3_)_y_Cs_0.4_FeSe was 0.6 K at ambient pressure as described in Ref. 5. The difference is in the symbols in graphs (Figure 1(c), Figures 3(a)–(d) and Figures 4(b) and (d)).

### Temperature dependence of *R*

The temperature dependence of *R* was measured in four-terminal measurement mode under pressure. The sample was introduced into the DAC in an Ar-filled glove box so as to apply the pressure to the sample without any exposure to air. The sample was loaded directly onto a plate of cubic BN/epoxy resin/rhenium in the DAC, and the four Pt electrodes for resistance measurement were between the sample and the plate. The experimental details for setting the sample in the DAC and for *R*-measurement are described elsewhere^20^. The pressure was determined by monitoring ruby fluorescence. A constant electric current, *I* ( = 100 μA), was supplied by an Advantest R6561 Multimeter, which also measured the voltage. To confirm that a reliable resistance measurement was obtained, the constant current flow in this measurement system and the linear *I* *vs.* *V* plot were checked frequently, implying that the data on resistance are reliable and the thermal voltage can be ignored.

## Supplementary Material

Supplementary Information Supplementary Information

## References

[b1] Ying T. P. *et al.* Observation of superconductivity at 30–46 K in A_x_Fe_2_Se_2_ (A = Li, Na, Ba, Sr, Ca, Yb, and Eu). Sci. Rep. 2, 426 (2012). 22645642 10.1038/srep00426 PMC3361284

[b2] Burrard-Lucas M. *et al.* Enhancement of the superconducting transition temperature of FeSe by intercalation of a molecular spacer layer. Nature Mater. 12, 15–19 (2013). 23104153 10.1038/nmat3464

[b3] Ying T. P. *et al.* Superconducting phases in potassium-intercalated iron selenides. J. Am. Chem. Soc. 135, 2951–2954 (2013). 23406203 10.1021/ja312705x

[b4] Sedlmaier S. J. *et al.* Ammonia-rich high-temperature superconducting intercalates of iron selenide revealed through time-resolved *in situ* X-ray and neutron diffraction. J. Am. Chem. Soc. 136, 630–633 (2014). 24354523 10.1021/ja411624q

[b5] Zheng L. *et al.* Superconductivity in (NH_3_)_y_Cs_0.4_FeSe. Phys. Rev. B 88, 094521 (2013).

[b6] Sakai Y. *et al.* Superconducting phases in (NH_3_)_y_*M*_x_FeSe_1−z_Te_z_ (*M* = Li, Na, and Ca). Phys. Rev. B 89, 144509 (2014).

[b7] Ye G. J. *et al.* Superconductivity in Yb_x_*M*_y_HfNCl (*M* = NH_3_ and THF). Phys. Rev. B 86, 134501 (2012).

[b8] Hsu F.-C. *et al.* Superconductivity in the PBO-type structure a-FeSe. Proc. Natl. Acad. Sci. USA 105, 14262 (2008). 18776050 10.1073/pnas.0807325105 PMC2531064

[b9] Margadonna S. *et al.* Pressure evolution of the low-temperature crystal structure and bonding of the superconductor FeSe (*T*_c_ = 37 K). Phys. Rev. B 80, 064506 (2009).

[b10] Fan D., Xu J., Ma M., Liu J. & Xie H. *P*-*V*-*T* equation of state of molybdenite (MoS_2_) by a diamond anvil cell and *in situ* synchrotron angle dispersive X-ray diffraction. Physica B 451, 53–57 (2014).

[b11] Guo J. G. *et al.* Superconductivity in the iron selenide K_x_Fe_2_Se_2_ (0 ≤ x ≤ 1.0). Phys. Rev. B 82, 180520(R) (2010).

[b12] Lee C.-H. *et al.* Effect of structural parameters on superconductivity in fluorine-free LnFeAsO_1−y_ (Ln = La, Nd). J. Phys. Soc. Jpn. 77, 083704 (2008).

[b13] Mizuguchi Y. & Takano Y. Review of Fe chalcogenides as the simplest Fe-based superconductor. J. Phys. Soc. Jpn. 79, 102001 (2010).

[b14] Sun L. L. *et al.* Re-emerging superconductivity at 48 kelvin in iron chalcogenides. Nature 483, 67–69 (2012). 22367543 10.1038/nature10813

[b15] Cai P. *et al.* Visualizing the microscopic coexistence of spin density wave and superconductivity in underdoped NaFe_1−x_Co_x_As. Nature Commun. 4, 1596 (2013). 23481404 10.1038/ncomms2592

[b16] Hatakeda T., Noji T., Kawamata T., Kato M. & Koike Y. New Li-ethylenediamine-intercalated superconductor Li_x_(C_2_H_8_N_2_)_y_Fe_2−z_Se_2_ with *T*_c_ = 45 K. J. Phys. Soc. Jpn. 82, 123705 (2013).

[b17] Ding X. *et al.* Influence of microstructure on superconductivity in K_x_Fe_2−y_Se_2_ and evidence for a new parent phase K_2_Fe_7_Se_8_. Nature Commun. 4, 1897 (2013). 23695691 10.1038/ncomms2913 PMC3674273

[b18] Guo J. *et al.* Pressure-driven quantum criticality in iron-selenide superconductors. Phys. Rev. Lett. 108, 197001 (2012). 23003077 10.1103/PhysRevLett.108.197001

[b19] Lu X. *et al.* Superconductivity in LiFeO_2_Fe_2_Se_2_ with anti-PbO-type spacer layers. Phys. Rev. B 89, 020507(R) (2013).

[b20] Shimizu K., Amaya K. & Suzuki N. Pressure-induced superconductivity in elemental materials. J. Phys. Soc. Jpn. 74, 1345–1357 (2005).

