# Supplementary material for: Emergence of double-dome superconductivity in ammoniated metal-doped FeSe
Source: Sci Rep. 2015 Apr 1;5:9477. doi: 10.1038/srep09477 (PMC4381328; doi:10.1038/srep09477)
Supplement: Supplementary Information [file srep09477-s1.pdf]

Supplementary Information

Emergence of double-dome superconductivity in ammoniated metal-doped FeSe

Masanari Izumi[1], Lu Zheng[1], Yusuke Sakai[1], Hidenori Goto[1], Masafumi Sakata[1,2,3,4], Yuki Nakamoto[4], Huyen L. T. Nguyen[1,2], Tomoko Kagayama[4], Katsuya Shimizu[4], Shingo Araki[5], Tatsuo C. Kobayashi[5], Takashi Kambe[5], Dachun Gu[6], Jing Guo[6], Jing Liu[7], Yanchun Li[7], Liling Sun[6], Kosmas Prassides[3,8], and Yoshihiro Kubozono[1,2*]

[1]Research Laboratory for Surface Science, Okayama University, Okayama 700-8530, Japan

[2]Research Centre of New Functional Materials for Energy Production, Storage and Transport, Okayama University, Okayama 700-8530, Japan

[3]Department of Chemistry, University of Durham, Durham DH1 3LE, UK

[4]Centre for Science and Technology under Extreme Conditions, Osaka University, Osaka 560-8531, Japan

[5]Department of Physics, Okayama University, Okayama 700-8530, Japan

[6]Institute of Physics and Beijing National Laboratory for Condensed Matter Physics, Chinese Academy of Science, Beijing 100190, China

[7]Institute of High Energy Physics, Chinese Academy of Science, Beijing 100049, China

[8]WPI Research Centre, Advanced Institute for Materials Research, Tohoku University, Sendai 980-8577, Japan

Table of Contents

1) *R* – *T* plots of the first $(NH_3)_yCs_{0.4}FeSe$ sample at 1.9, 5.0, 7.0 and 11 GPa
2) T_c is plotted as a function of α in the pressure range of 0 - 13 GPa.
3) T_c is plotted as a function of anion height in the pressure range of 0 - 13 GPa.
4) *R* – *T* plots of the second $(NH_3)_yCs_{0.4}FeSe$ sample at 8.8, 9.5, 11 and 14 GPa
5) T_c is plotted as a function of anion height in the pressure range of 0 - 41 GPa.

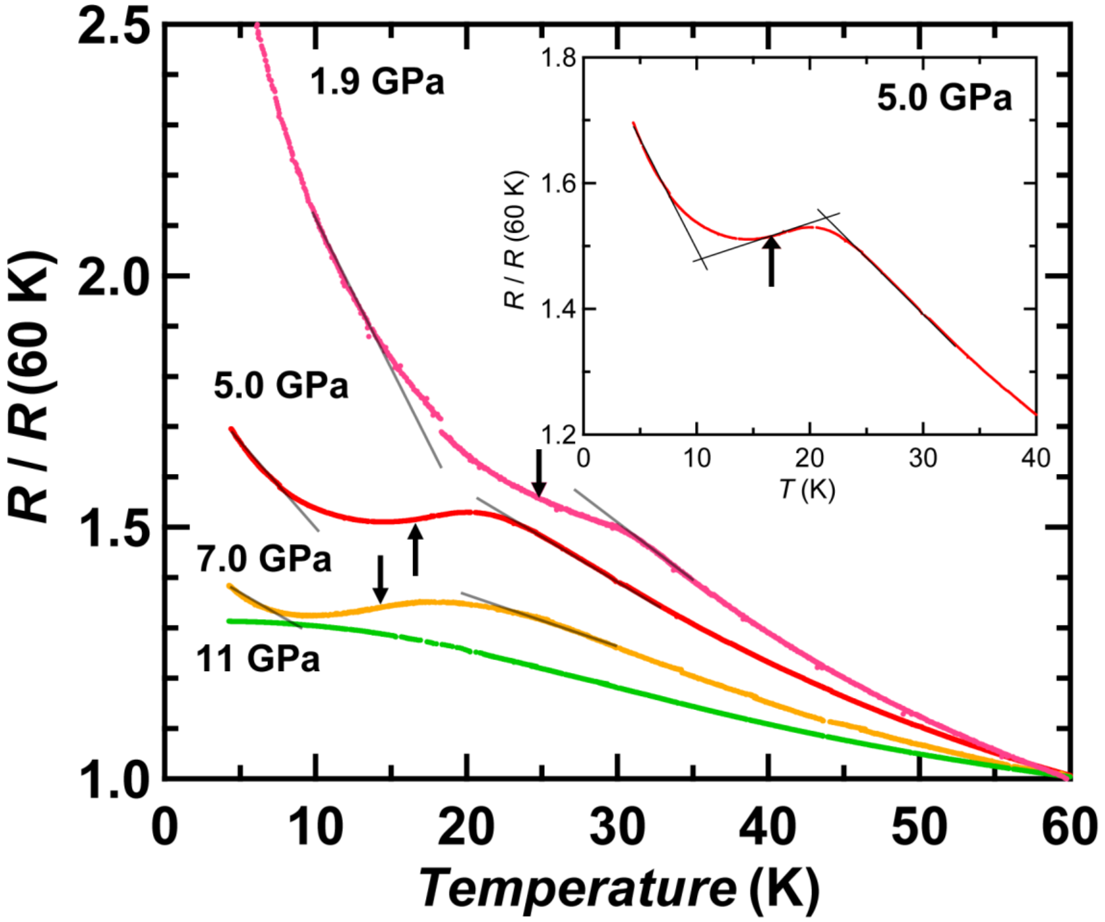

Figure S1. Resistance *vs.* temperature plots of the first $(NH_3)_yCs_{0.4}FeSe$ sample at 1.9, 5.0, 7.0 and 11 GPa. The grey solid lines refer to the fitting ones for the normal and superconducting states. The arrows correspond to the T_c's determined from the midpoint. As described in text, the drop is not observed at 11 GPa. Inset: the expanded resistance *vs*. temperature plot at 5.0 GPa, which is provided to show how to determine the T_c.

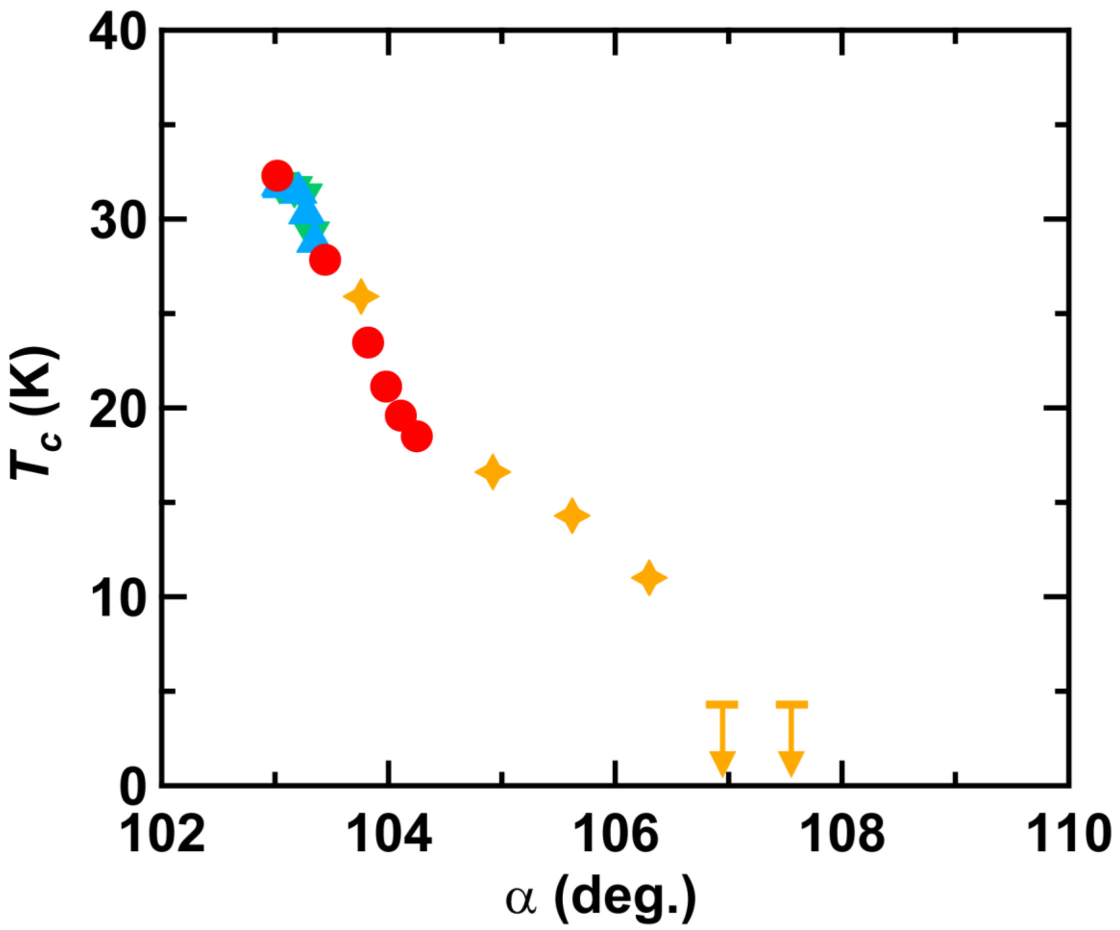

Figure S2: T_c is plotted as a function of α. The symbols are defined in the caption of Figure 1(c) in text. The arrows indicate T_c's lower than the temperatures denoted by bars.

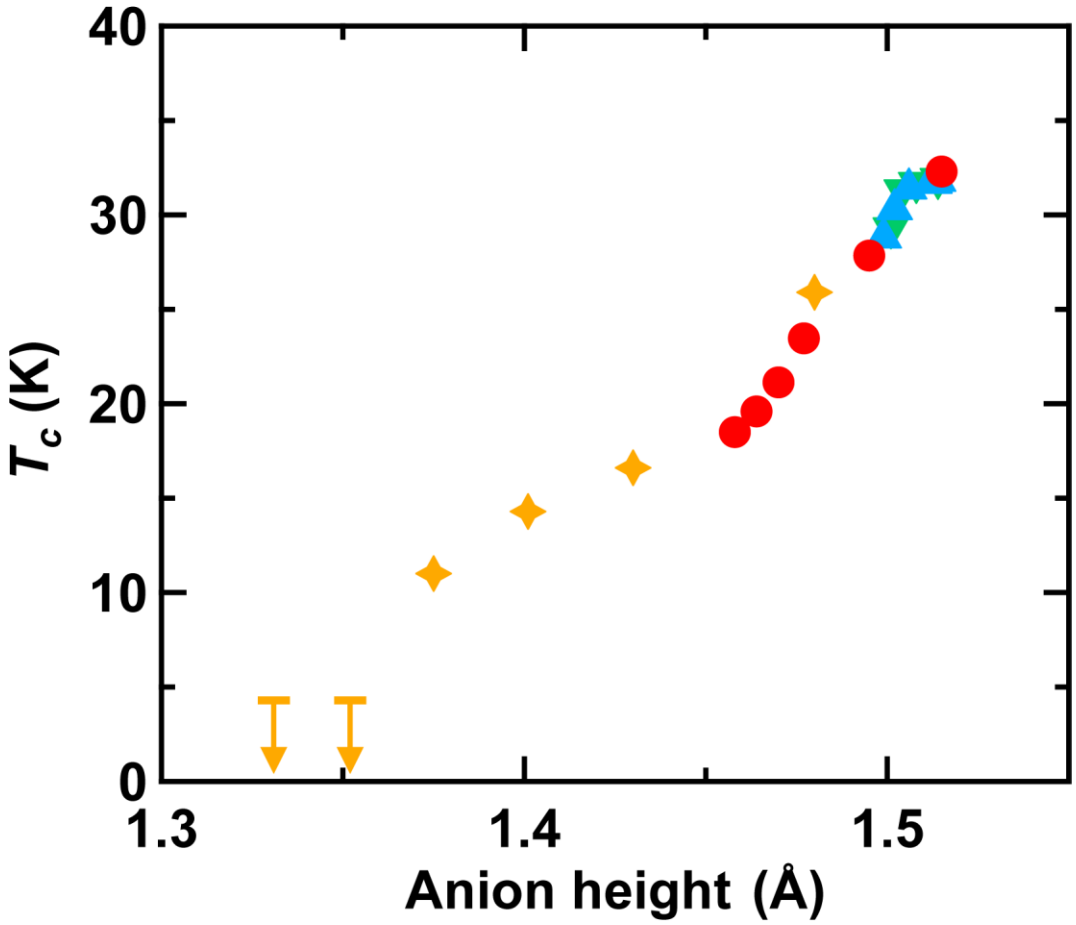

Figure S3: T_c is plotted as a function of anion height in the pressure range of 0- 13 GPa. The symbols are defined in the caption of Figure 1(c) in text. The arrows indicate T_c's lower than the temperatures denoted by bars.

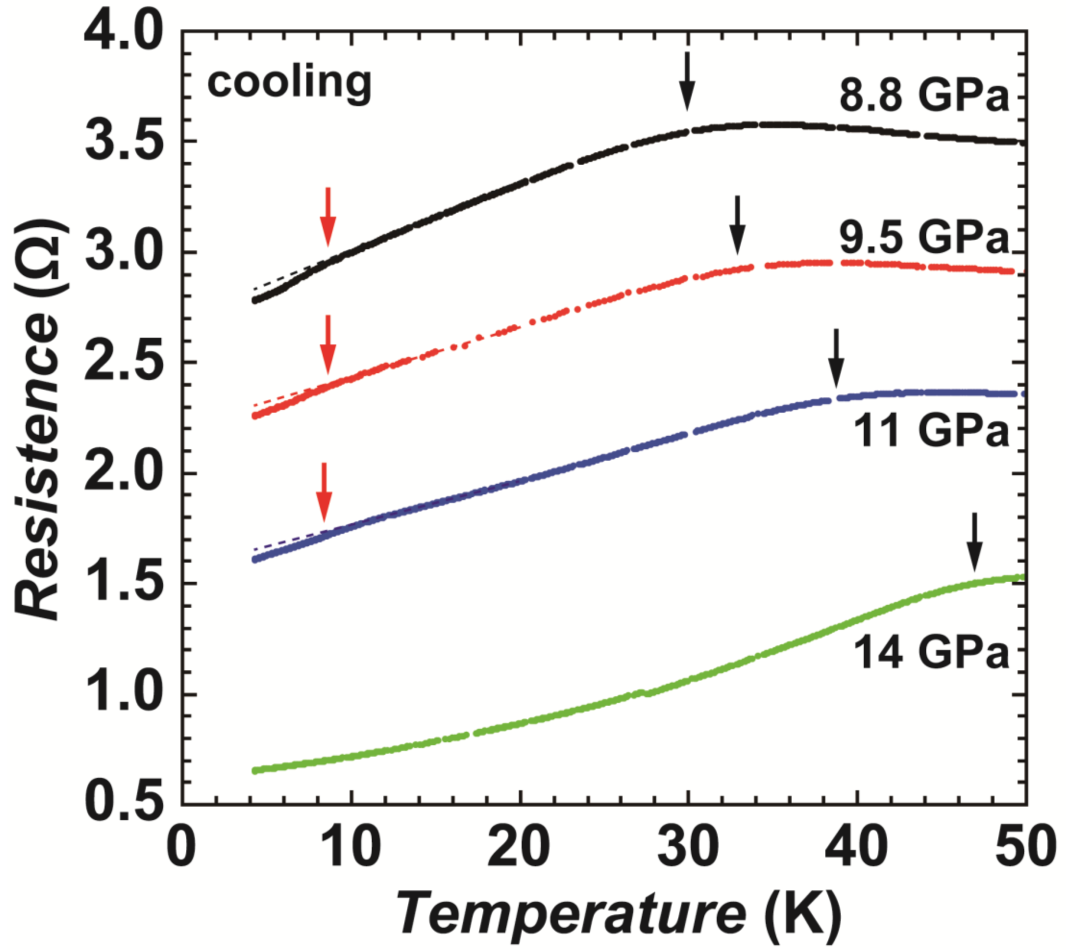

Figure S4: Resistance *vs.* temperature plots of the second $(NH_3)_yCs_{0.4}FeSe$ sample at 8.8, 9.5, 11 and 14 GPa. The arrows correspond to the T_c's in two phases. As described in text, small drops are observed in *R* – *T* plots below 10 K at 8.8 and 9.5 GPa. The drop is observed even at 11 GPa, but no drop is observed below 10 K at 14 GPa.

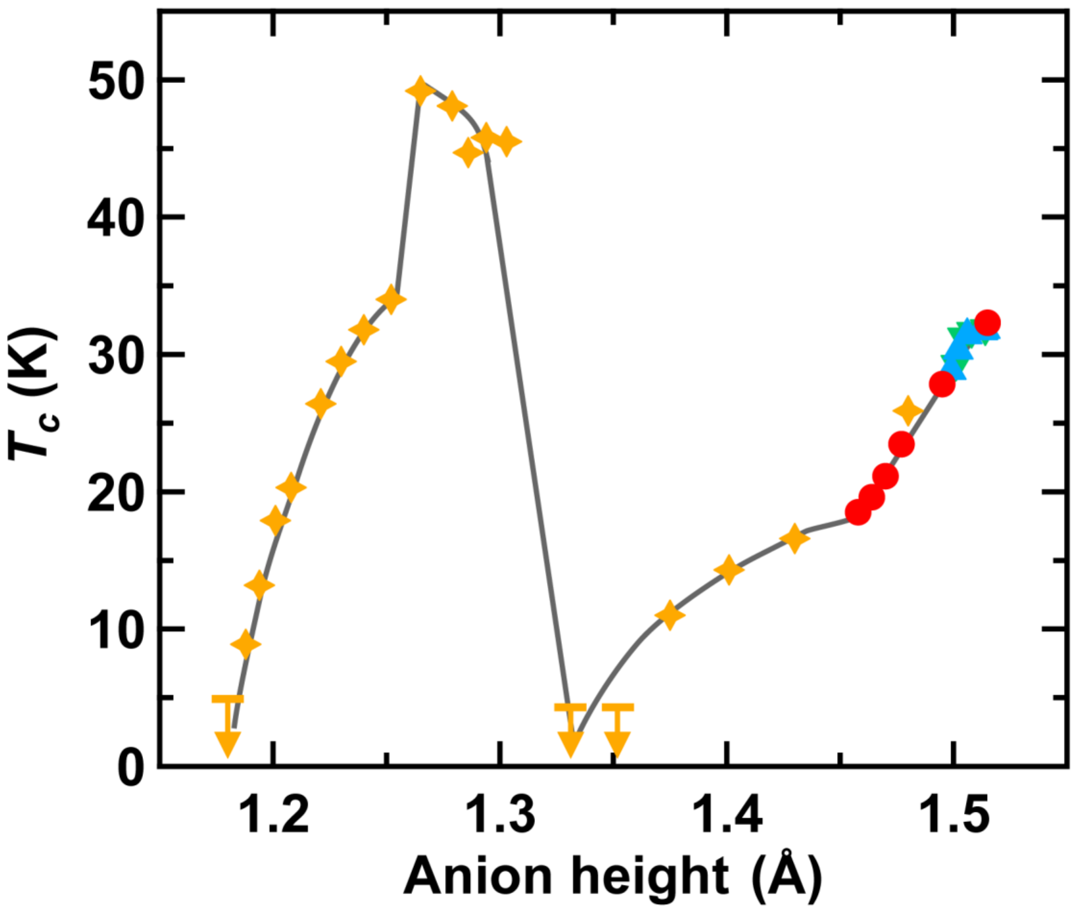

Figure S5: T_c is plotted as a function of anion height in the pressure range of 0 - 41 GPa. The symbols are defined in the caption of Figure 1(c) in text. The arrows indicate T_c's lower than the temperatures denoted by bars. The solid line is a visual aid.
